# Effects of running distance on per-step and cumulative lower-extremity loading during a simulated treadmill half marathon

**DOI:** 10.3389/fpubh.2026.1741450

**Published:** 2026-01-21

**Authors:** Wenjing Quan, Huiyu Zhou, Yiwen Ma, Datao Xu, Zixiang Gao, Xuting Wang, Zsolt Radak, Yaodong Gu

**Affiliations:** 1 Faculty of Sports Science, Ningbo University, Ningbo, China; 2 Human Performance Laboratory, Faculty of Kinesiology, University of Calgary, Calgary, AB, Canada; 3 Research Institute of Sport Science, Hungarian University of Sport Science, Budapest, Hungary

**Keywords:** Achilles tendon, cumulative damage, long-distance running, patellofemoral joint, running musculoskeletal injuries

## Abstract

**Background:**

The cumulative damage impulses have been proposed as a crucial parameter for analyzing joint kinetics and tissue loading during prolonged running. Although prolonged running may have detrimental effects, research focusing on the bones and soft tissues of the lower extremity remains limited. Therefore, this study aimed to examine how treadmill half-marathons influence biomechanical variables in high-performing endurance runners. We additionally quantified per-step peak load, impulse, cumulative impulse, and cumulative weight impulse of the Achilles tendon (AT) and the patellofemoral joint (PFJ) during half marathon running.

**Method:**

Sixteen high-performing endurance runners (9 males and 7 females) completed a half marathon on a Zebris FDM-T pressure-sensing treadmill in a standardized biomechanics laboratory. The lower extremity kinematic and kinetic parameters were measured at every 10 km (0 km, 10 km, and 20 km) and subsequently processed using Visual3D and simulation musculoskeletal modeling. We used a one-way repeated measures ANOVA to determine the main effect of running distance on the outcome variables.

**Results:**

During the running stance phases, contact time and step frequency significantly increased from 10 km to 20 km (*p* < 0.005). Conversely, step length and stride length exhibited a significant decrease (*p* < 0.005). Ankle peak plantarflexion angle, ankle push-off phase range of motion (ROM), and hip braking phase ROM were significantly reduced at 10 km (*p* < 0.005). The cumulative loading on the ankle, knee, and hip joints was significantly greater at both 10 km and 20 km (*p* < 0.005). The AT force and cumulative weighted impulse value decreased at 10 km (*p* < 0.005) but significantly increased at 20 km running (*p* < 0.005). Simultaneously, the PFJ cumulative loading parameter was increased from 10 km to 20 km running (*p* < 0.005).

**Conclusion:**

As the running distance increases, the running mechanism is significantly altered. The cumulative loading on the knee and hip significantly increased, indicating a shift in the compensatory mechanism from the proximal to the distal joint. Cumulative loading on the AT and PFJ increased with the accumulated running distance, which directly reflects that prolonged running might cause greater cumulative loading on the lower extremity.

## 1 Introduction

Running is one of the most easily accessible forms of exercise and fitness, which can enhance physical fitness and prevent chronic diseases (1). Recently, there has been a significant increase in participation in half marathons or 10 km races compared to full marathons (2). While running offers numerous health benefits, over 40% of runners experience overuse injuries related to the activity, predominantly affecting the lower limbs (3). Epidemiological studies indicate that runners engaged in high-intensity or endurance activities, such as marathon training and competitions, exhibit a higher incidence of running-related injuries (4). Runners commonly experience various injuries, including knee pain, tibial stress fractures and Achilles tendinopathy (3, 5). Overuse injury refers to an injury caused by excessive loading or damage to a tendon or bone, which results in microdamage but does not further damage the tissue structure (6, 7). Without sufficient time for tissue remodeling, the accumulation of microdamage significantly increases the risk of lower-limb overuse injuries (8). Thus, understanding how mechanical loading at common injury sites responds to running acute distances can help improve running performance and injury prevention.

The biomechanical risk factors leading to overuse injuries from prolonged distance running result in alterations in lower-limb joint kinematics, kinetic parameters, and cumulative loading at injury sites (9–11). Studies have shown that with increased running distance, the contact time and stride frequency significantly increase, while stride length decreases (12, 13). Previous studies have demonstrated that after fatigue running significantly increases the ankle dorsiflexion angle, knee flexion angle, and hip range of motion (ROM) (14, 15). Moreover, research indicates that post-fatigue running may decrease knee joint stiffness and cause a significant increase in knee flexion angle (16). After a half marathon, runners often suffer fatigue, and there are repetitive cumulative damages induced in the body that may increase the risks for musculoskeletal overuse injuries. Repetitive submaximal loading induces damage, which causes a degradation of the material properties of the tissue. Structural failure may thereafter occur as a result of this weakened condition. These injuries may be represented as a mechanical fatigue process (7, 17). Failure, especially progressive failure, makes tendon and bone overuse injuries more likely over time. Studies have indicated that the fatigue lives of human tendons exhibit a stronger inverse power law (18, 19). Previous research has quantified cumulative loading at common sites of running injuries, such as the tibia, AT, and PFJ, during the stance phase of running (8, 10, 20). Higher cumulative loading would increase the risk of running injuries. However, it remains unclear how cumulative loading affects the lower extremity, particularly at the PFJ and AT, during prolonged running.

Cumulative load is a crucial parameter for assessing joint kinetics and tissue loading during long-distance running. A previous study has reported that the locations of the tibia, PFJ, and AT are the most common running-related injury sites for the runners (21). After prolonged running, runners frequently experience discomfort or pain in the knee, particularly associated with PFJ injuries (22). Patellofemoral pain syndrome is characterized by increased stress and contact forces at the PFJ, which can impair athletic performance and potentially induce a fear of movement (23). Individuals suffering from PFJ injuries demonstrate increased shear joint stress compared to healthy controls (24). Furthermore, running a prolonged distance has been shown to alter knee kinematics and kinetic parameters. For example, joint work may shift from the distal (ankle) to proximal (knee and hip) joints following a fatigue run (25, 26). An increase in mechanical work at the knee joint leads to heightened musculotendinous forces, subsequently elevating knee joint contact forces. Additionally, a previous study indicated that following a fatigue run, the PFJ force significantly increased, potentially exacerbating patellofemoral pain and raising the risk of knee joint injuries (11).

During the prolonged running, the AT experiences substantial repetitive loads (27). It has been shown that there are no significant changes in AT strain in recreational runners after 30 min of running (28), whereas another study reported a significant reduction in tendon stiffness following 90 min of running (29). Additionally, Stanley et al. indicated that the AT can adapt to the high-volume training to absorb and release the energy during prolonged running for highly trained distance runners (30). Furthermore, a previous study revealed that high-intensity running to fatigue may significantly reduce the peak AT force (11). Nevertheless, the relationship between long-distance running and cumulative damage to the AT is still not fully understood. Considering that the initial peak longitudinal strain is a key predictor of tendon fatigue life (18), the application of a weighting factor could provide a valuable method for assessing cumulative tendon damage during a half-marathon and estimating the associated risk of AT injury.

High-performing endurance runners are characterized by their frequent training and competition at faster speeds compared to recreational runners (31). Previous studies have defined high-performing endurance runners; for example, competitive runners are reported to complete a 10 km race in approximately 35 min (32). Padulo et al. indicated that elite runners finish a full marathon with an average time of around 2 h and 21 min (33). Research has shown that high-performing endurance runners achieve faster speeds and improved performance through increased step lengths and flight times (33, 34). Furthermore, it has been established that Achilles tendinopathy is associated with training at higher speeds, while patellofemoral pain correlates with increased training volumes (35, 36). Stephen et al. demonstrated that high-performing endurance runners—defined as those completing a 10 km race in less than 32 min for males and less than 36 min for females—exhibit higher loading rates on the AT and PFJ with increased running speed during the stance phase (37). High-performing endurance runners often experience repetitive loading during high-intensity or endurance activities, which can diminish joint mechanical stability and neuromuscular control, consequently increasing the risk of overuse injuries (38). It is essential to investigate the cumulative changes in the lower limbs of high-performing endurance runners during prolonged running. Therefore, based on the definition provided by Stephen et al., this study aims to investigate how the cumulative changes occur during a half marathon for these runners.

The current knowledge of cumulative weight impulses to lower-limb bones and tendons during prolonged running in the high-performing endurance runners remains insufficient. Although previous research has primarily focused on immediate biomechanical responses following a run, there is limited knowledge regarding the changes in these parameters at various stages of a prolonged run. Therefore, the present study aimed to investigate the effects of accumulated running distance on lower extremity kinematics and kinetics at specific distance checkpoints (0 km, 10 km, and 20 km) and cumulative loading on the PFJ and AT in high-performing endurance runners. We hypothesized that a treadmill half marathon may have distinct effects on lower extremity kinematics and kinetics parameters. We expected that contact time and step frequency would increase, lower extremity joint stability would decrease, and cumulative loading on the PFJ and AT might be higher as the distance accumulated during the half marathon.

## 2 Method

### 2.1 Participants

Sixteen high-performing endurance runners (mean ± SD; age: 21.00 ± 1.31 years; height: 172.00 ± 7.13 cm; body mass: 61.64 ± 8.72 kg) participated in the present study, comprising 9 male and 7 female runners. Sample size was determined using G*Power (version 3.1.9.2, University of Kiel, Germany) for a repeated-measures ANOVA, with the following parameters: significance level (*α*) = 0.05, statistical power (1 − *β*) = 0.75, and effect size (Cohen’s *f* = 0.25) (39, 40). The primary outcome variables were selected based on a prior study that reported statistically significant differences in Achilles tendon loading during the running stance phase (41). Detailed participant information is presented in Table 1. Recruitment was conducted via social media platforms and posters distributed at the university and local running clubs. All participants met the following inclusion criteria: aged 18–35 years; running at least three times per week; completion of at least two half-marathon competitions in the past 6 months; at least 40 km running per week (37); a habitual rearfoot strike pattern; having achieved a self-reported 10-km personal best time of less than 32 min (males) or 36 min (females) (34); and no lower extremity injuries in the past 6 months. This study protocol received approval from the Ningbo University Human Ethics Committee (TY2024034), and all participants provided informed consent.

**Table 1:** Demographic, training and performance characteristics of high-performing endurance runners.

Parameters	Total	Male	Female
Number (*n*)	16	9	7
Age (years)	21.00 ± 1.41	20.80 ± 1.30	21.50 ± 2.12
Height (cm)	172.00 ± 7.70	175.20 ± 6.61	164.00 ± 1.41
Body mass (kg)	61.64 ± 9.15	67.57 ± 5.00	51.25 ± 1.71
Running experience (years)	7.63 ± 2.00	7.67 ± 2.35	7.57 ± 1.62
Weekly mileage (km)	53.31 ± 9.94	57.78 ± 11.21	47.57 ± 3.31
10 km PB time (min)	33.50 ± 2.01	31.79 ± 0.15	35.70 ± 0.20

### 2.2 Experiment protocol

**Figure 1:**
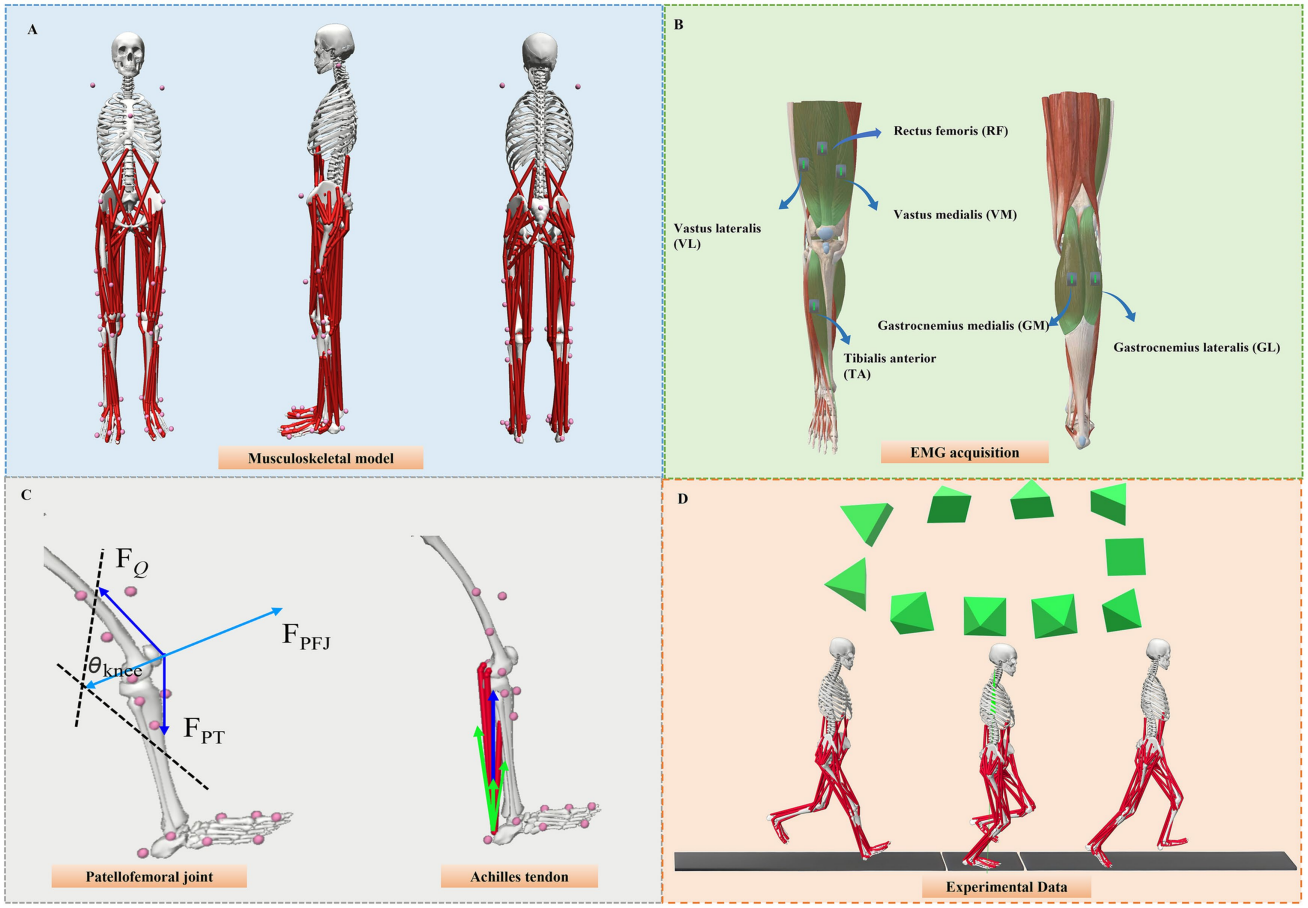
Provides an overview of the research workflow employed in this study. **(A)** This figure depicts the placement of the reflective marker on the lower extremity. **(B)** The illustration highlights the placement of the surface EMG tests on the rectus femoris (RF), vastus medialis (VM), vastus lateralis (VL), gastrocnemius medialis (GM), gastrocnemius lateralis (GL), and tibialis anterior (TA). **(C)** The patellofemoral joint experiences compressive stress between the femur and patella, which is influenced by the force generated by the quadriceps muscles (F_Q_) and the knee flexion angle (Ɵ _knee_). This interaction alters both the angle of pull between the quadriceps and patellar tendons as well as the contact area. The force vector of the patellar tendon (FPT) is indicated, while FPFJ represents the contact force between the femur and patella. The force vector of the Achilles tendon (illustrated by the blue arrow) reflects the cumulative effect of the forces exerted by the individual triceps surae muscles, specifically the soleus and the lateral and medial gastrocnemius, as shown by the green arrows. **(D)** The Vicon motion system cameras and force plate during the running data collection process.

Prior to testing, all participants performed a 10-min warm-up on the treadmill at 10 km/h and familiarized themselves with the experimental protocol. Participants wore their appropriate running shoes and spandex tights during the data collection. A total of 38 reflective markers (14 mm diameter) were affixed bilaterally on the lower limbs and trunk (Figure 1A). The lower-body marker set included five anatomical segments: pelvis, bilateral thighs, shanks, feet, and torso (42). All markers were placed individually, except for four T-shaped frames on the thighs and calves, each consisting of three tracking points. Furthermore, all reflective markers were attached with skin-medical tape that prevented them from dropping off during the whole running protocol.

Moreover, we used the wireless 32-channel surface electromyogram (EMG) system (Delsys, Boston, MA, USA) electrodes to collect the EMG signals at a sampling of 1,000 Hz during the running stance phases. Skin preparation was performed in accordance with SENIAM guidelines. We placed the electrodes on the rectus femoris (RF), vastus medialis (VM) and lateralis (VL), gastrocnemius medialis (GM) and lateralis (GL), and tibialis anterior (TA) (Figure 1B) to collect the muscle activation during the half marathon running (43). Before the running trials, maximal voluntary contractions (MVC) were measured in all participants (43). A motion capture system consisting of 10 cameras (Oxford Metrics Ltd., Oxford, UK) and two force plates (Kistler Type 9,281 B, Kistler Instrument AG, Winterthur, Switzerland) was synchronized to collect marker trajectories and ground reaction force (GRF) data at 200 Hz and 1,000 Hz, respectively (26, 43) (Figure 1D).

The data collection started with a static calibration trial for model simulation. Participants then ran along a 15-meter overground runway at 4.0 ± 0.5 m/s to obtain pre-running kinematic and GRF data during the stance phase. Thereafter, participants completed the half-marathon running task at a constant speed of 14 km/h on the Zebris FDM-T treadmill system (Zebris Medical GmbH, Isny, Germany). Spatiotemporal parameters were obtained directly from the treadmill, which continuously records right-foot contact and toe-off phases (41). For each running distance (pre-running, 10 km, and 20 km), 30 s of treadmill data were collected for spatiotemporal analysis. After completing 10 km and 20 km on the treadmill, participants returned to the 15-meter overground runway, where synchronized kinematic and GRF data were collected again. Each participant completed four successful running trials through the embedded force plate. The successful trials are defined as the participants running with the entire right foot placed on the force plate at 4.0 ± 0.5 m/s for each participant. Additionally, smart speed devices (Smart Speed, Fusion Sport Inc., Burbank, CA, USA) were positioned on either side of the track to monitor running velocity (43). The running biomechanics trials were conducted 3 times in total: pre-running, post-10 km running, and post-20 km running.

### 2.3 Data analysis

Raw marker trajectories were labelled in Vicon Nexus 1.8.5 (Vicon, Metrics Ltd., Oxford, UK), and the C3D files of the running stance phase for the lower limbs were subsequently exported. All the collection data were then imported into Visual3D (V6.0, C-Motion, Germantown, MD, USA) to calculate joint kinematics and kinetics variables (42). Kinematic data were low-pass filtered at 20 Hz, and ground reaction forces were low-pass filtered at 50 Hz, in accordance with previous studies (41). Raw EMG signals were processed using a zero-lag, fourth-order Butterworth band-pass filter (20–450 Hz) to remove motion artifacts and high-frequency noise. The linear envelopes of the EMG signals were then normalized to the peak value obtained during the maximal voluntary contraction (MVC) for each muscle (43). All EMG signals were then time-normalized to 100 points (1–100%) to allow comparison with simulated muscle activation profiles (43). All biomechanical variables for each stance phase were time-normalized to 101 points utilizing MATLAB software (R2024a; MathWorks, Natick, MA, USA).

Joint angles of the ankle, knee, and hip were computed using Cardan angles, with the distal segment relative to the proximal segment in Visual 3D software. The joint moments were calculated using a standard inverse dynamics approach (44), and all kinetic variables were normalized according to each participant’s body mass. Analyses focused on the running stance phase of the right leg, defined as the period from initial ground contact to toe-off, during which the value of ground reaction force exceeded 20 N (43). The range of motion (ROM) for the hip, knee, and ankle joints was also compared during the braking and push-off phases (45). Ankle dorsiflexion ROM was defined as the interval from initial contact to peak dorsiflexion, and ankle plantarflexion ROM as the interval from peak dorsiflexion to toe-off. Knee flexion ROM was defined from initial contact to peak knee flexion, and knee extension ROM from peak knee flexion to toe-off. Hip flexion ROM was defined from initial contact to peak hip flexion, and hip extension ROM from peak hip flexion to toe-off (46). Angular impulse was determined as the time integral of joint moment during the loading and push-off phases of stance (47).

Cumulative impulse ( $I_{C}$ ):


$$I_{C}=n\times I$$

The total number of strides required to complete a 1,000-m distance was denoted as *n*. For each joint, the cumulative load was calculated as the product of the stance-phase angular impulse in the dominant moment direction and the stride count (*n*) necessary to cover 1,000 m (48, 49). In addition, we quantified the peak joint moment per stance phase, as well as the kinematic parameters, including peak joint angles and ROM of the ankle, knee, and hip joints during the braking and push-off phases.

### 2.4 Musculoskeletal model

The customized musculoskeletal analysis was conducted in OpenSim (Version 4.3, Stanford, CA, USA), which has been widely used to estimate muscle forces and joint contact force during the running stance phases. The 2,392 musculoskeletal model, which comprises 10 rigid body segments, 23 degrees of freedom, and 92 musculotendon units (42), was utilized for all analyses. Marker trajectories exported from Visual3D in “osim” format were imported into OpenSim for musculoskeletal simulation, and the model was scaled for each participant based on anthropometric measurements. Firstly, inverse kinematics (IK) and inverse dynamics (ID) tools were conducted to calculate the joint angle and joint moment. Then, we used the static optimization (SO) tool to compute the lower limb muscle forces during the running stance phases. The analysis tool was applied to compute the patellofemoral contact force during the running stance phases (43). The patellofemoral contact stress was estimated by dividing the compressive component of the joint contact force by the contact area at each knee flexion angle (Figure 1C). Contact area values were obtained from sex-specific MRI-based measurements reported by Besier et al. (50).

The AT force was calculated as the sum of the forces generated by the soleus and the medial and lateral gastrocnemius muscles (Figure 1C) (42). Changes in AT length were estimated by dividing the tendon force by the tendon stiffness, which had previously been measured via ultrasound at 420 N·mm^−1^ in earlier studies (20, 51). The AT strain was calculated based on previous research using a formula relative to the resting tendon length, which was defined as 250 mm (20).

Simultaneously, we compared the peak stress and strain values per step for the AT and the PFJ, which reflect cumulative loading. Using the number of steps required to cover 1 km, we estimated the CD by applying a weighted impulse for PFJ stress, AT force, and AT strain, as demonstrated in Equation 1. This method, proposed by Firminger et al., incorporates a tissue-dependent weighting factor to estimate kilometer-specific loading for running distances of 0 km, 10 km, and 20 km (8).


Cumulative−weight impulse=n[∫titf(xs)bdt]1b

In this study, *n* denotes the number of right-foot contacts, tᵢ represents the start time of the running stance phase, $t_{f}$ the end time of the stance phase, and X_s_ the measure of internal bone peak impulse loading. The parameter b is a weighting factor that reflects the slope of the power function relating fatigue life to tissue-specific stress or strain. For bone, b was set to 7, and for tendon tissue it was set to 9, based on previous studies (8, 18, 52, 53). The weighted impulse approach emphasizes that load magnitude is a more critical determinant of cumulative tissue strain than the number of loading cycles. The step count per kilometer was calculated by taking the inverse of the stride duration (1/stride duration), which reflects the total number of right-foot contacts included in the cumulative strain analysis of both bone and tendon tissues.

### 2.5 Statistics analyses

All biomechanical parameters at the three running distances were presented as means and standard deviations. Statistical analyses were performed using SPSS version 25.0 (SPSS Science, Chicago, IL, USA). Data normality was assessed with the Shapiro–Wilk test. One-way repeated-measures ANOVAs with Bonferroni corrections for multiple comparisons were used. The level of significance was set at 0.05.

## 3 Results

### 3.1 Spatiotemporal parameters

A significant main effect of running distance was observed on contact time (*F* = 43.396, *p* < 0.001, partial *η^2^* = 0.41), step length (*F* = 10.791, p < 0.001, partial *η^2^* = 0.15), step frequency (*F* = 9.523, *p* = 0.003, partial *η^2^* = 0.13), and stride length (*F* = 12.250, *p* < 0.001, partial *η^2^* = 0.16) across various running distances (Table 2). Specifically, contact time increased significantly with running distance. At 20 km, the contact time was significantly greater than at 0 km (*p* < 0.001) and 10 km (*p* < 0.001). Additionally, after 10 km of running, contact time was significantly longer than at 0 km (*p* = 0.003). Step frequency also increased significantly with distance, with values at 10 km (*p* < 0.001) and 20 km (*p* < 0.001) being higher than at 0 km. Additionally, the step frequency was significantly increased at 20 km (*p* = 0.002) in comparison to the 10 km running stance phases. In contrast, step length significantly decreased at 20 km (*p* < 0.001) and 10 km (*p* < 0.001) in comparison to 0 km. Finally, stride length exhibited a significant decrease with increasing running distance. A significant reduction was observed at 20 km compared to 10 km (*p* = 0.008).

**Table 2:** Spatiotemporal metrics during the different distances running stance phases.

Parameters	0 km	10 km	20 km	F-value	*p*-value	ES partial η^2^
Contact time (s)	0.217 ± 0.013	0.224 ± 0.023	0.241 ± 0.011	43.396	**<0.001**	0.41
Step length (cm)	115.93 ± 2.60	113.82 ± 3.80	112.99 ± 4.49	10.791	**<0.001**	0.15
Step frequency (Steps/min)	175.99 ± 6.68	177.17 ± 7.02	179.71 ± 4.29	9.523	**0.003**	0.13
Stride length (cm)	229.13 ± 8.85	227.24 ± 9.12	223.57 ± 7.52	12.250	**<0.001**	0.16

Significant differences are indicated in bold (*p* < 0.05).

### 3.2 Joint angle

**Figure 2:**
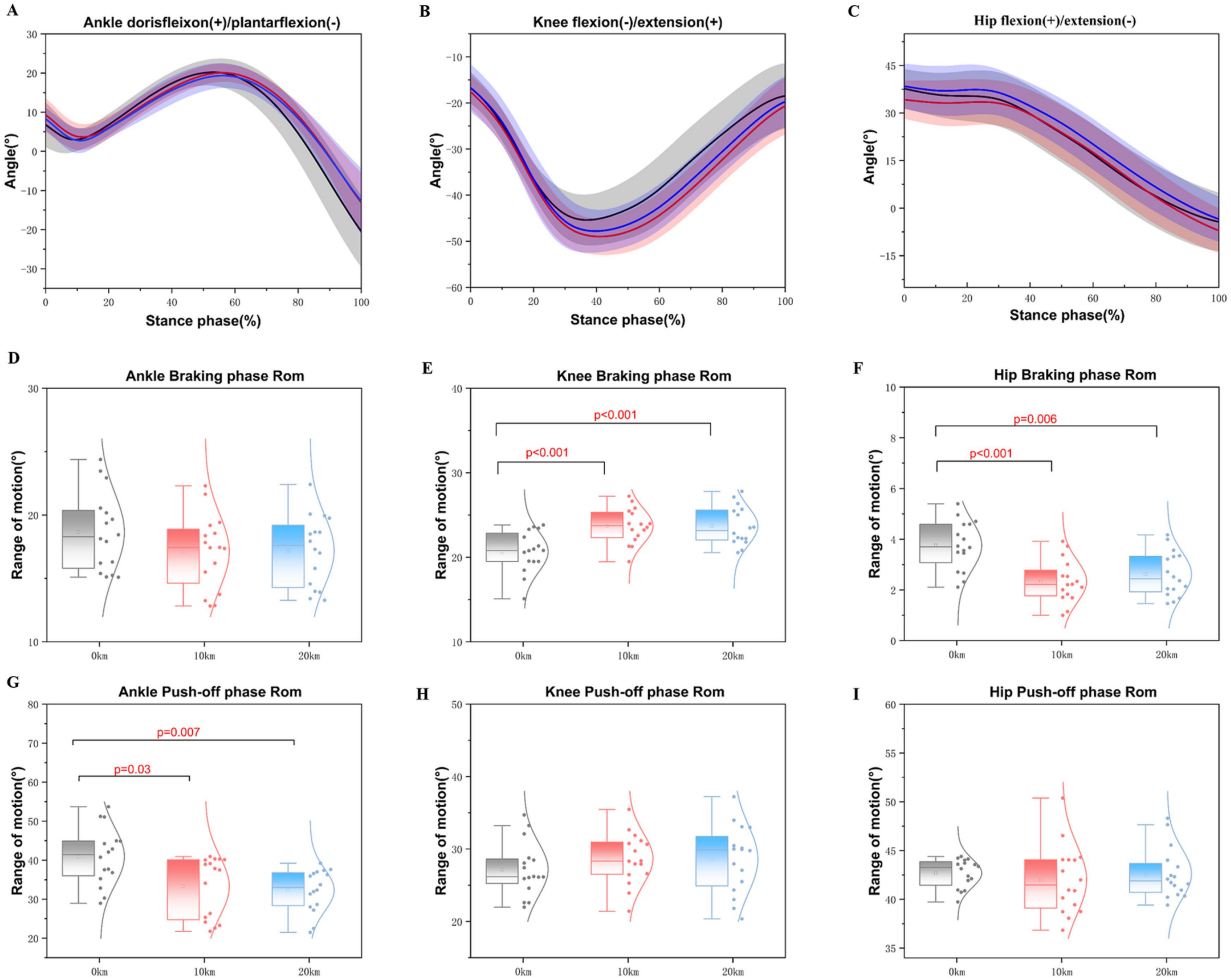
Illustrates the changes in the ankle, knee, and hip joint angles in the sagittal plane and range of motion (ROM) of the barking and push-off phases during the running stance phase of a half marathon. **(A–C)** Mean values of ankle joint angles, knee joint angles, and hip joint angles during the running stance phases of the half marathon for all the participants. **(D–F)** Mean values represent the ankle, knee, and hip joint range of motion during the braking phases of a half marathon. **(G–I)** Represents the values of ankle, knee, and hip joint range of motion during the push-off phases of a half marathon.

A main effect of running distance was observed on the ankle push-off phase ROM (*F* = 7.052, *p* = 0.004, partial *η^2^* = 0.32), knee ROM during the braking phase (*F* = 16.120, *p* < 0.001, partial *η^2^* = 0.52), and hip ROM during the braking phase (*F* = 10.842, *p* < 0.001, partial *η^2^* = 0.42) (Table 3; Figure 2). The ankle ROM during the push-off phase was significantly greater at 0 km compared to 10 km (*p* < 0.001) and 20 km (*p* = 0.003) (Figure 2H). Conversely, the hip ROM during the braking phase significantly declined at 10 km (*p* < 0.001) and 20 km (*p* = 0.006) compared to 0 km (Figure 2F). In contrast, the knee ROM during the braking phase significantly increased at 10 km (*p* < 0.001) and 20 km (*p* < 0.001) compared to 0 km (Figure 2E). There were no significant changes observed in the ankle ROM during the braking phase (*F* = 1.421, *p* = 0.258, partial *η^2^* = 0.09), knee push-off ROM (*F* = 1.596, *p* = 0.223, partial *η^2^* = 0.10), or hip push-off ROM (*F* = 0.292, *p* = 0.669, partial *η^2^* = 0.02).

**Table 3:** Mean ± standard deviation joint ROM angle and peak angle value for each running distance.

Parameters	0 km	10 km	20 km	F-value	*p*-value	ES partial η^2^
Range of motion (°)						
Ankle braking phases ROM	18.65 ± 3.07	17.12 ± 2.92	17.19 ± 2.78	1.421	0.258	0.09
Ankle push-off phases ROM	40.95 ± 7.36	33.29 ± 7.74	32.33 ± 5.45	7.052	**0.004**	0.32
Knee braking phases ROM	20.66 ± 2.41	23.58 ± 2.08	23.70 ± 2.26	16.120	**<0.001**	0.52
Knee push-off phases ROM	27.19 ± 3.69	28.65 ± 3.51	28.60 ± 4.68	1.596	0.223	0.10
Hip braking phases ROM	3.76 ± 0.96	2.34 ± 0.84	2.62 ± 0.88	10.842	**<0.001**	0.42
Hip push-off phases ROM	42.68 ± 1.47	42.01 ± 3.52	42.61 ± 2.60	0.292	0.669	0.02
Peak angle (°)						
Peak ankle dorsiflexion angle	20.52 ± 2.95	20.22 ± 2.26	19.56 ± 3.10	2.074	0.143	0.03
Peak ankle plantarflexion angle	−20.43 ± 9.15	−13.07 ± 7.66	−12.82 ± 8.32	25.374	**<0.001**	0.29
Peak knee flexion angle	−45.66 ± 5.42	−49.34 ± 3.89	−45.32 ± 4.56	11.677	**<0.001**	0.16
Peak hip flexion angle	38.35 ± 5.36	34.94 ± 6.38	39.15 ± 7.38	8.563	**<0.001**	0.12
Peak hip extension angle	−4.33 ± 9.24	−7.06 ± 7.04	−3.46 ± 7.12	4.942	**0.009**	0.07

Statistically significant differences are indicated in bold (*p* < 0.05).

A significant effect of running distance was shown on the peak ankle plantarflexion angle (*F* = 25.374, *p* < 0.001, partial *η^2^ =* 0.29), peak knee flexion angle (*F* = 11.677, *p* < 0.001, partial *η^2^* = 0.16), peak hip flexion angle (*F* = 8.563, *p* < 0.001, partial *η^2^* = 0.12), and peak hip extension angle (*F* = 4.942, *p* = 0.009, partial *η^2^* = 0.07) during the half marathon (Table 3; Figure 2). Specifically, the peak ankle plantarflexion angle at 20 km (*p* < 0.001) and 10 km (*p* < 0.001) showed a significant reduction compared to 0 km (Figure 2A). As running distance increased, the peak knee flexion angle significantly increased at 10 km (*p* < 0.001) compared to 0 km; however, after 20 km, the peak knee flexion angle significantly decreased (*p* = 0.004) compared to 0 km (Figure 2B). For the hip angles, the peak hip flexion angle significantly increased at 10 km compared to both 0 km (*p* = 0.002). The peak angle of hip flexion was significantly greater during the 20 km than the 10 km (*p* < 0.001). On the contrary, the peak hip extension angle was significantly increased during the 10 km rather than the 0 km (*p* = 0.024) and 20 km (*p* = 0.003) running stance phases (Figure 2C). No significant changes were observed in peak ankle dorsiflexion (*F* = 2.074, *p* = 0.143, partial *η^2^* = 0.03) during the half marathon running.

### 3.3 Joint moment

**Figure 3:**
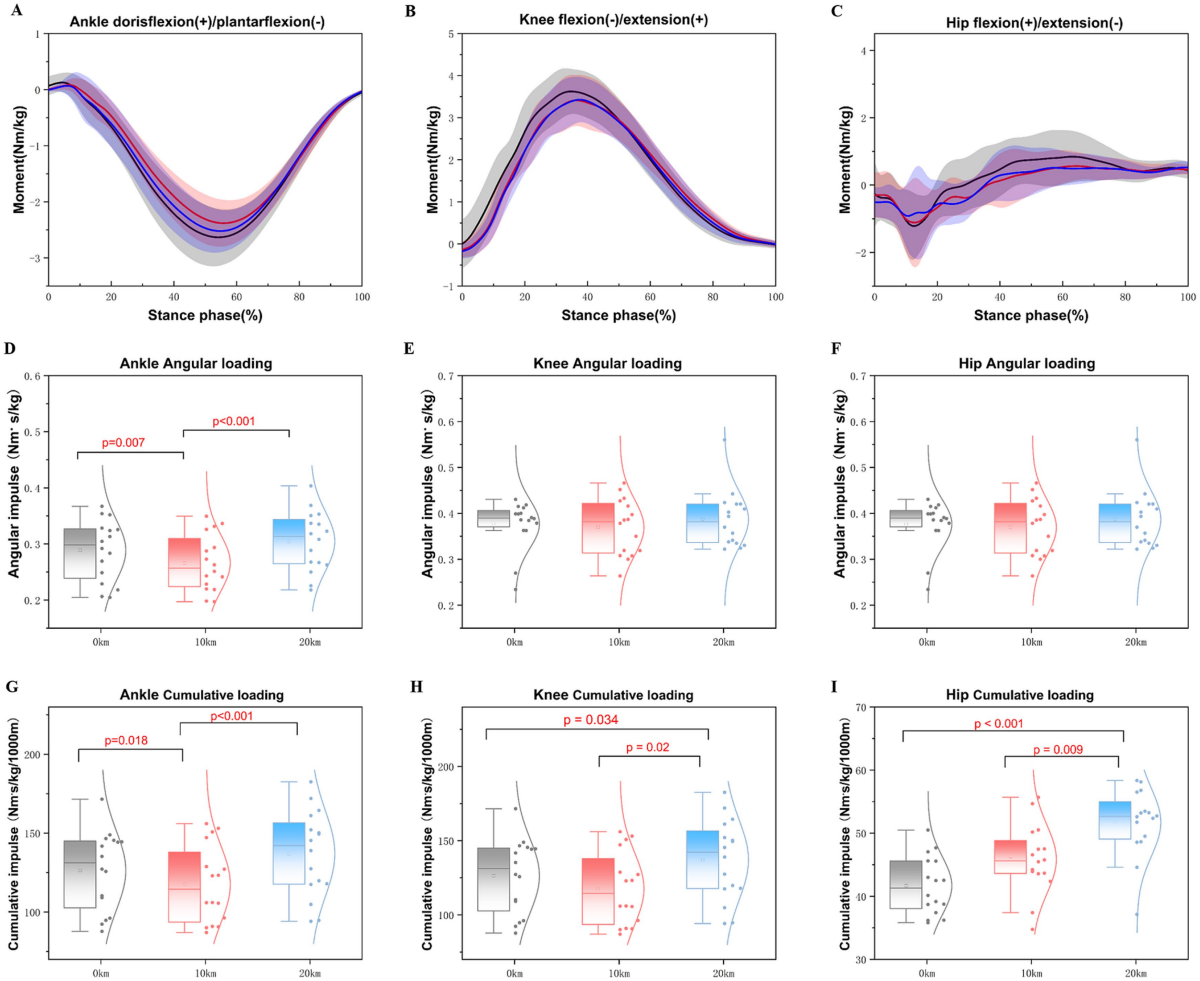
Illustrates the changes in the ankle, knee, and hip joint moment in the sagittal plane during the running stance phase of a half marathon. **(A–C)** Mean values of ankle joint moment, knee joint moment, and hip joint moment during the running stance phases of the half marathon for all the participants. **(D–F)** Mean values of ankle, knee, and hip joint peak angular loading during the running stance phases of the half marathon for all the participants. **(G–I)** Meaning values of ankle, knee, and hip joint peak cumulative loading during the running stance phases of the half marathon for all the participants.

Significant differences were observed in joint moments, moment impulses, and cumulative loading variables during half marathon running (Table 4; Figure 3). A main effect of distance was noted on the peak ankle plantarflexion moment (*F* = 7.241, *p* = 0.002, partial *η^2^* = 0.10) and the peak hip flexion moment (*F* = 12.561, *p* < 0.001, partial *η^2^* = 0.17). The results indicated a significant decrease in the peak ankle plantarflexion moment at 10 km (*p* < 0.001) compared to 0 km (Figure 3A). Furthermore, as the distance increased, the peak ankle plantarflexion moment significantly decreased during the 20 km (*p* = 0.029) compared to the 10 km distance (Figure 3A). Similarly, the peak hip flexion moment was significantly reduced (*p* < 0.001) at both 10 km and 20 km compared to 0 km (Figure 3C). Nonetheless, no significant differences were observed in the peak moments of ankle dorsiflexion (*F* = 1.518, *p* = 0.223, partial *η^2^* = 0.02), knee flexion moment (*F* = 4.620, *p* = 0.013, partial *η^2^* = 0.07), knee extension moment (*F* = 0.930, *p* = 0.435, partial *η^2^* = 0.01), or hip extension moment (*F* = 0.211, *p* = 0.723, partial *η^2^* = 0.01) during the half marathon.

**Table 4:** Mean ± SD ankle, knee and hip peak moment, moment impulse and cumulative loading value for each running distance.

Parameters	0 km	10 km	20 km	F-value	*p*-value	ES partial η^2^
Peak moment (N/kg)						
Peak ankle dorsiflexion moment	0.17 ± 0.16	0.13 ± 0.10	0.16 ± 0.12	1.518	0.223	0.02
Peak ankle plantarflexion moment	−2.67 ± 0.50	−2.40 ± 0.41	−2.54 ± 0.38	7.241	**0.002**	0.10
Peak knee flexion moment	3.67 ± 0.56	3.45 ± 0.63	3.48 ± 0.56	4.620	**0.013**	0.07
Peak knee extension moment	−0.25 ± 0.18	−0.21 ± 0.15	−0.23 ± 0.19	0.830	0.435	0.01
Peak hip flexion moment	1.28 ± 0.65	0.90 ± 0.45	0.87 ± 0.42	12.561	**<0.001**	0.17
Peak hip extension moment	−1.75 ± 0.54	−1.82 ± 0.74	−1.75 ± 0.73	0.211	0.723	0.01
Stance phase angular impulse (Nm^.^s/kg)						
Ankle plantarflexion	0.29 ± 0.06	0.27 ± 0.05	0.30 ± 0.06	6.485	**0.004**	0.09
Knee extension	0.38 ± 0.06	0.37 ± 0.07	0.39 ± 0.07	1.681	0.193	0.03
Hip flexion	0.12 ± 0.07	0.10 ± 0.04	0.12 ± 0.04	1.455	0.238	0.02
Cumulative loading (Nm^.^s/kg/1000 m)						
Ankle plantarflexion	126.36 ± 28.17	117.62 ± 25.38	137.05 ± 29.93	7.181	**0.003**	0.10
Knee extension	164.23 ± 25.38	162.48 ± 30.83	173.44 ± 31.50	4.242	**0.020**	0.06
Hip flexion	41.67 ± 315.51	45.86 ± 15.11	51.72 ± 13.70	7.538	**0.001**	0.11

Statistically significant differences are indicated in bold (*p* < 0.05).

### 3.4 Joint angular impulse and cumulative impulse

Table 4 shows the angular impulse and cumulative impulse mechanics of the ankle, knee, and hip joints in the sagittal plane during half-marathon running. The results revealed a significant decrease in the ankle plantarflexion angular impulse at 10 km (*p* = 0.007) compared to 0 km (*F* = 6.485, *p* = 0.004, partial *η^2^* = 0.09) during the running stance phases (Figure 3D). However, the angular impulse for ankle plantarflexion significantly increased at 20 km when compared to 10 km (*p* < 0.001) (Figure 3D). There were no significant changes observed in the knee flexion stance phase angular impulse (*F* = 1.681, *p* = 0.193, partial *η^2^* = 0.03) (Figure 3E) or the hip flexion stance phase angular impulse (*F* = 1.455, *p* = 0.238, partial *η^2^* = 0.02) (Figure 3F). Additionally, the cumulative impulse for ankle plantarflexion (*F* = 7.181, *p* = 0.003, partial *η^2^* = 0.10) significantly increased with distance, showing a significant difference at 20 km compared to 0 km (*p* < 0.001) (Figure 3G). Conversely, the cumulative impulse for ankle plantarflexion significantly decreased at 10 km (*p* = 0.018) compared to 0 km as the distances increased (Figure 3G). The knee flexion cumulative impulse also increased significantly with distance (*F* = 4.242, *p* = 0.020, partial *η^2^* = 0.06), indicating that the knee flexion cumulative impulse was greater at 20 km compared to both 0 km (*p* = 0.034) and 10 km (*p* = 0.002) during the running stance phases (Figure 3H). Furthermore, the hip flexion cumulative impulse significantly increased with distance (*F* = 7.538, *p* = 0.001, partial *η^2^* = 0.11), demonstrating that the hip cumulative impulse was greater at 20 km compared to 0 km (*p* < 0.001) and 10 km (*p* = 0.009) during the running stance phases (Figure 3I).

### 3.5 Model validation

**Figure 4:**
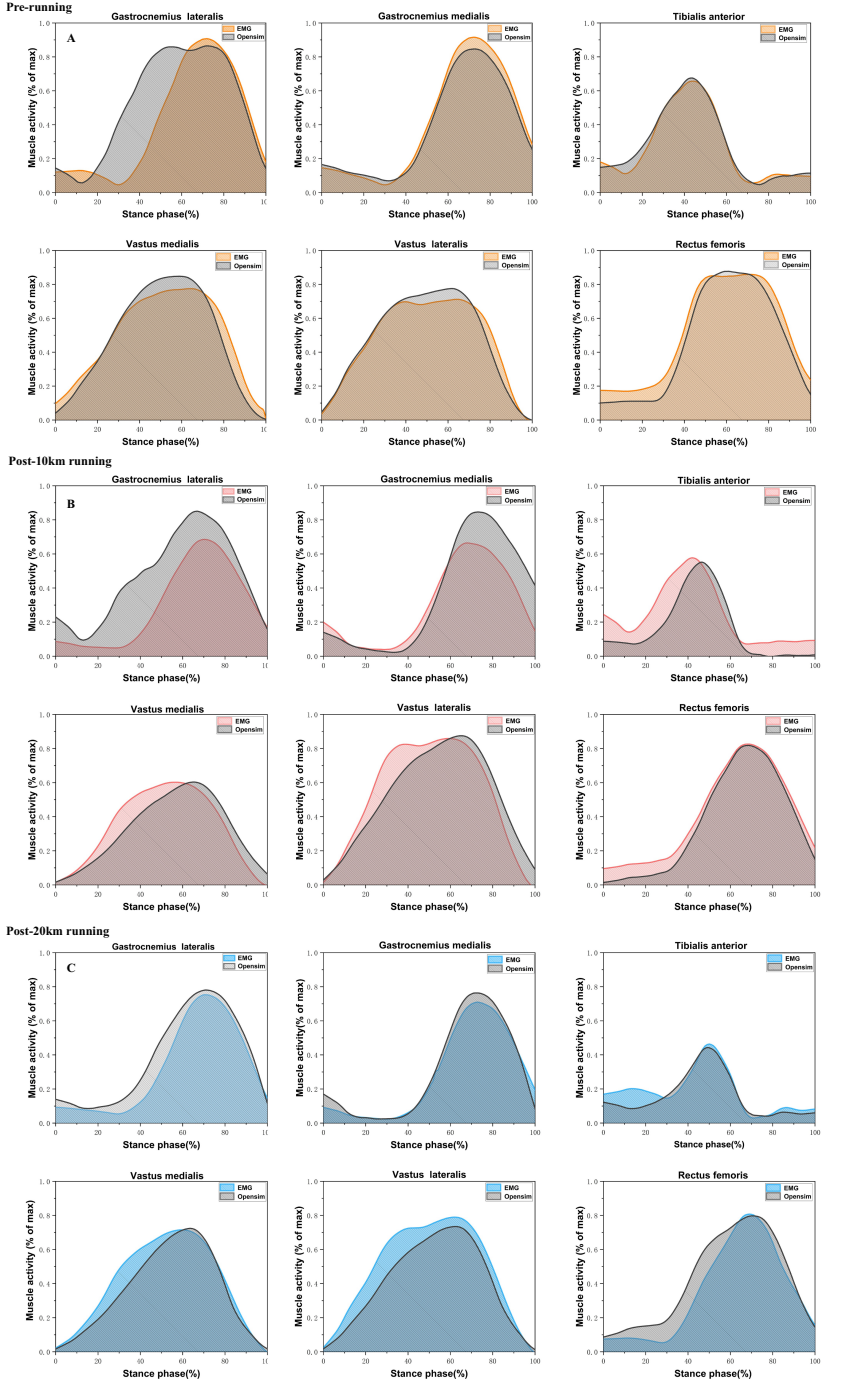
Comparison of experimentally measured EMG muscle activation and musculoskeletal model–simulated activation of the rectus femoris (RF), vastus medialis (VM), vastus lateralis (VL), gastrocnemius medialis (GM), gastrocnemius lateralis (GL), and tibialis anterior (TA) during the running stance phase **(A)** pre-running; **(B)** post-10 km running; **(C)** post-20 km running.

To verify the validity of the musculoskeletal model, we compared the time- and magnitude-normalized EMG data to the modeled muscle activation. Figure 4 illustrates a comparison of muscle activation results acquired from the EMG sensor with those simulated by OpenSim across different running distances. The results indicated that the activation levels of the six selected muscles were predominantly consistent, demonstrating that the musculoskeletal model established in this study exhibits a significant level of reliability.

### 3.6 Achilles tendon force and Achilles tendon strain

**Figure 5:**
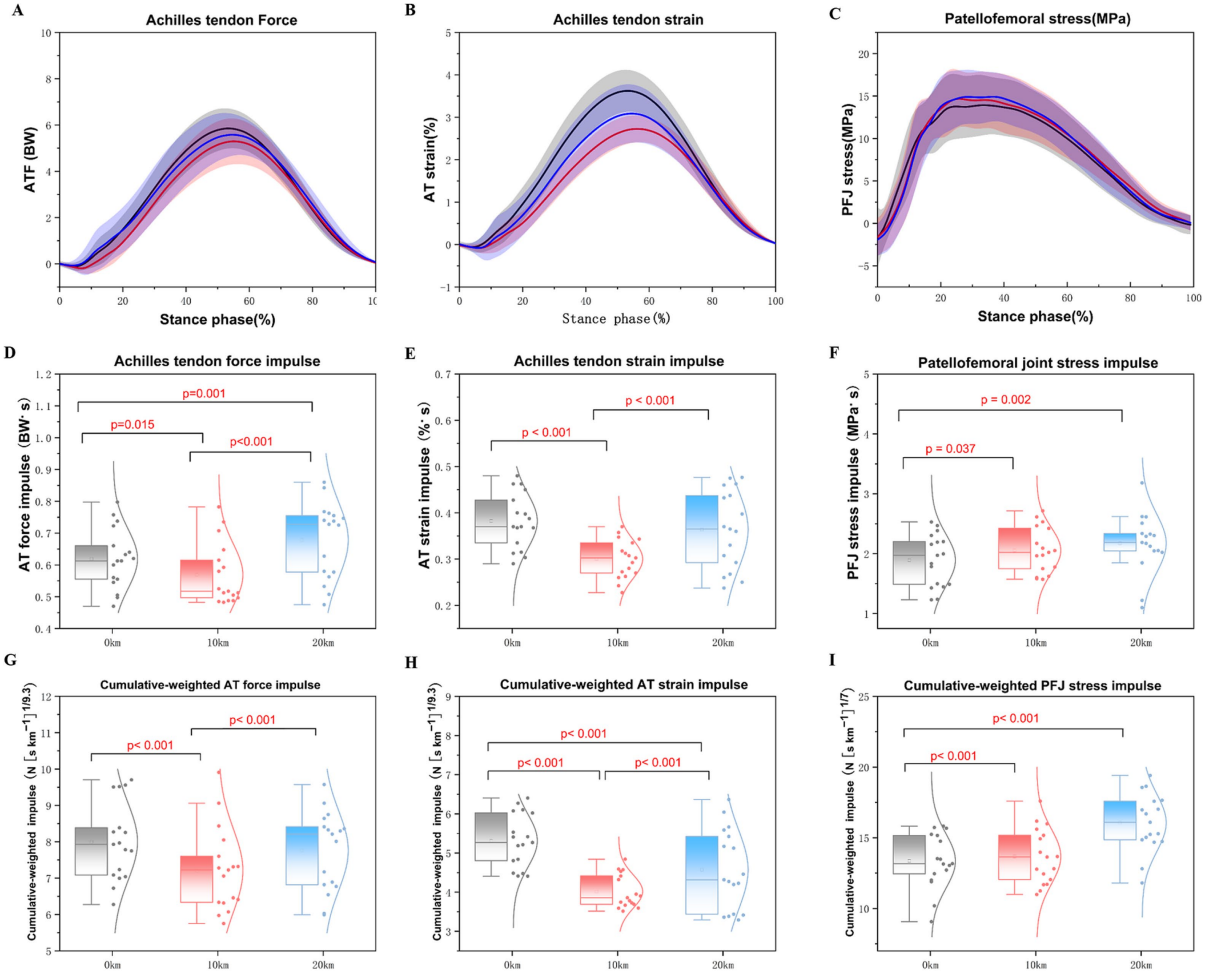
Changes in the Achilles tendon force, Achilles tendon strain, and patellofemoral joint during the running stance phase of a half marathon. **(A–C)** Mean values of Achilles tendon force, Achilles tendon strain, and patellofemoral stress changes during the running stance phase of a half marathon. **(D–F)** Mean values of the peak impulse of Achilles tendon force, Achilles tendon strain, and patellofemoral joint stress. **(G–I)** Mean the value of peak cumulative weight impulse of Achilles tendon force, Achilles tendon strain, and patellofemoral joint stress per step in the half marathon running.

The main effects of running distance on peak force, peak strain, force impulse, strain impulse, cumulative weight force impulse, and cumulative weight strain impulse for the AT were identified (Table 5; Figure 5). A significant main effect of running distance was observed on the AT peak force (*F* = 6.711, p = 0.002, partial *η^2^* = 0.10), AT peak strain (*F* = 56.523, *p* < 0.001, partial *η^2^* = 0.47), AT peak force impulse (*F* = 14.276, *p* < 0.001, partial *η^2^* = 0.19), and AT peak strain impulse (*F* = 30.005, *p* < 0.001, partial *η^2^* = 0.32) during the half marathon. The peak AT force significantly diminished at 10 km (*p* < 0.001) in comparison to 0 km. Conversely, the AT force significantly increased after 20 km (*p* = 0.021) when compared to 10 km (Figure 5A). The AT peak strain exhibited a significant decrease with increasing distances, particularly at 10 km (*p* < 0.001) and 20 km (*p* < 0.001) during the running stance phases compared to 0 km (Figure 5B). However, at 20 km, the AT peak strain significantly increased (*p* < 0.001) compared to 10 km. Regarding the AT force impulse, a significant decrease was noted at 10 km compared to 0 km (*p* = 0.015) (Figure 5D). The AT force impulse was significantly greater at 20 km compared to both 0 km (*p* = 0.011) and 10 km (*p* < 0.001) during the running stance phases (Figure 5D). Additionally, a significant reduction in AT peak strain impulse was found at 10 km compared to both 0 km (*p* < 0.001) and 20 km (*p* < 0.001) (Figure 5E). There were significant main effects of distance on the cumulative weight AT force impulse (*F* = 6.415, *p* = 0.003, partial *η^2^* = 0.09) and the cumulative AT strain impulse (*F* = 51.638, *p* < 0.001, partial *η^2^* = 0.45) (Figure 5G). The results indicated that with increased running distance, the cumulative weight AT force impulse significantly declined during the 10 km run compared to 0 km (*p* = 0.001) and 20 km (*p* = 0.005) (Figure 5G). The cumulative AT strain impulse significantly decreased during the 10 km (*p* < 0.001) and 20 km (*p* < 0.001) runs compared to 0 km (Figure 5H). Furthermore, the cumulative AT strain impulse significantly increased at 20 km (*p* < 0.001) compared to 10 km (Figure 5H).

**Table 5:** Mean ± SD deviation Achilles tendon force, strain, impulse, cumulative weighted impulse patellofemoral joint stress, impulse, cumulative weighted impulse values for each running distance.

Parameters	0 km	10 km	20 km	F-value	*p*-value	ES partial η^2^
AT peak force (BW)	5.91 ± 0.85	5.32 ± 0.98	5.65 ± 0.93	6.711	**0.002**	0.10
AT peak strain (%)	3.66 ± 0.48	2.75 ± 0.33	3.11 ± 0.69	56.523	**<0.001**	0.47
Patellofemoral peak stress (MPa)	14.97 ± 3.75	15.59 ± 3.50	15.91 ± 3.26	1.495	0.229	0.02
AT peak force impulse (BW^.^s)	0.62 ± 0.11	0.57 ± 0.11	0.68 ± 0.15	14.276	**<0.001**	0.19
AT peak strain impulse(%^.^s)	0.38 ± 0.07	0.30 ± 0.05	0.37 ± 0.09	30.005	**<0.001**	0.32
Patellofemoral peak stress impulse (Mpa^.^s)	1.92 ± 0.50	2.07 ± 0.44	2.22 ± 0.57	6.645	**0.003**	0.10
Cumulative-weighted AT force impulse (N [s km^−1^]^1/9.3^)	7.98 ± 1.18	7.22 ± 1.33	7.79 ± 1.33	6.415	**0.003**	0.09
Cumulative-weighted AT strain impulse (% [s km^−1^]^1/9.3^)	5.32 ± 0.72	4.03 ± 0.48	4.60 ± 1.03	51.638	**<0.001**	0.45
Cumulative-weighted Patellofemoral impulse (MPa [s km^−1^]^1/7^)	13.24 ± 3.00	13.76 ± 2.77	15.92 ± 4.03	14.428	**<0.001**	0.19

Statistically significant differences are indicated in bold (*p* < 0.05).

### 3.7 Patellofemoral joint

The main effects of running distance on peak stress, stress impulse, and CD stress impulse were observed in the PFJ during the half marathon (Table 5; Figure 5). Specifically, both the peak stress impulse (*F* = 6.645, *p* = 0.003, partial *η^2^* = 0.10) and the CD of the PFJ impulse (*F* = 14.428, *p* < 0.001, partial *η^2^* = 0.19), demonstrated significant differences throughout the half- marathon. As the distance increased, the peak PFJ stress impulse was significantly greater at 10 km (*p* = 0.037) and 20 km (*p* = 0.002) compared to 0 km (Figure 5F). Furthermore, the CD of PFJ stress was also significantly greater at 10 km (*p* < 0.001) and 20 km (*p* < 0.001) in comparison to 0 km (Figure 5I). However, no significant differences were found in the PFJ peak stress variables across the three checkpoint distances (*F* = 1.495, *p* = 0.229, partial *η^2^* = 0.02) (Figure 5C).

## 4 Discussion

In the present study, we investigated how a treadmill half marathon influences lower extremity running mechanics and the cumulative damage loading of the PFJ and AT. We hypothesized that a treadmill half marathon would demonstrate altered lower extremity biomechanical properties. Our findings indicated that half marathon running changes the gait pattern, characterized by increased contact time and step frequency, along with a gradual increase in peak knee flexion and a decrease in peak ankle plantarflexion angle. Furthermore, as the running distance increased, the ROM during the ankle push-off phase and the hip braking phase was reduced, while cumulative loading on the ankle, knee, and hip joints increased. However, cumulative loading on the AT decreased at 10 km and subsequently increased at 20 km, whereas cumulative loading on the PFJ increased significantly following the half marathon. These biomechanical changes may reflect movement strategy adaptations caused by the neuromuscular fatigue during a treadmill half marathon.

Researchers have found that half marathons generally alter running gait patterns and spatiotemporal parameters. Our results are consistent with previous studies, demonstrating an increase in contact time and step frequency, alongside a decrease in both step length and stride length during the half marathon running (12, 13, 54). During the half marathon running, these spatiotemporal changes reflect a fatigue-related adjustment in running mechanics, which may negatively affect running performance. In addition, the altered gait pattern may represent a movement strategy to adjust posture while running with neuromuscular fatigue (55). Runners appear to increase contact time and reduce stride length as a compensatory strategy to maintain postural stability and accommodate fatigue-related constraints on forward propulsion (56). In the present study, runners ran at a fixed running speed on the treadmill for all conditions, and an increase in step frequency necessarily corresponded to a decrease in step length.

During prolonged running, it is noteworthy that half marathon running alters the lower extremity kinematics and kinetics. These parameters can contribute to changing the running mechanism, potentially influencing a running overuse injury (57). We observed a significant decrease in ankle ROM at push-off phases and the peak ankle plantarflexion angle at 10 km and 20 km of running. With the increased running distance, the repetitive loading on the lower extremity may cause plantar flexor fatigue and decreased ankle stability during the running stance phases (26). These results were consistent with a previous study indicating that running-induced fatigue affects the ankle plantarflexion angle during the push-off phases (58). It has been demonstrated that the lower ankle plantarflexion angle during the push-off phases can lead to a higher peak propulsive force to improve the running performance (59). Thus, during the half marathon, high-performing endurance runners would decrease their ankle plantarflexion angle to improve propulsive force, consistent with the previous study. The present study observed a significant decrease in the ankle plantarflexion moment after both 10 km and 20 km running and no significant difference in the ankle dorsiflexion angle during the half marathon running. Quan et al. have found that running-induced fatigue increases the ankle joint’s total work, and positive work was lower than pre-running (26). The decline in elastic energy release after running fatigue can explain this phenomenon. The results from post-10 km and post-20 km running showed an increase in both ankle plantarflexion angular impulse and cumulative loading. The magnitude of ankle loading can influence the cumulative loading on the foot tissues, which can lead to mechanical fatigue and increase the risk of plantar fasciitis (60, 61).

The present study observed a significant increase in peak knee flexion angle and knee ROM at the braking phases during a treadmill half marathon. These results align with a previous study that indicated a significant increase in maximum knee flexion after a half marathon (15, 55). Following half-marathon running, prolonged exertion can induce neuromuscular fatigue (62). Concurrently, the activation of calf muscles was decreased, particularly the medial gastrocnemius, lateral gastrocnemius, and soleus muscles (63). While the muscle activation decreased, the knee joint must absorb more energy to maintain forward body propulsion, which can lead to increased knee flexion (4). Our study found that running a half marathon on a treadmill might lead to a significant increase in knee extension cumulative loading. After prolonged running, joint work shifts from the ankle to the knee and hip joints, which can increase knee joint loading during the stance phases. Simultaneously, the observed increase in knee flexion angle and cumulative loading on the knee may potentially cause knee pain and elevate the risk of patellofemoral pain (64).

After completing a half marathon, there were significant alterations in the hip mechanism. During running, the hip joint plays a crucial role in lower limb motion (26). Our findings revealed a significant reduction in peak hip flexion angle and hip braking phase range of motion (ROM) at 10 km and 20 km compared to 0 km. These alterations indicate that a lower hip flexion angle reduces propulsion efficiency during the running stance phases. Furthermore, our findings suggest that, with increased running distance, the cumulative loading on the hip joint significantly rises in the sagittal plane. These changes align with a previous study that reported a significant increase in hip vertical excursion following fatigue during running, which may elevate repetitive loading and predispose runners to overuse injuries (65).

The results of the present study indicate a reduction in AT loading variables after 10 km but significantly greater after 20 km of running. High-performing endurance runners have higher training volume and competitive speeds (31), which may cause higher cumulative loading in the AT (37). Research demonstrates a significant incidence and prevalence of Achilles tendinopathy among competitive runners (21). In this study, we observed a higher cumulative loading value on the AT after 20 km, providing evidence that elite long-distance runners are at an increased risk for AT injuries. Previous research has demonstrated that acute high-intensity running can diminish peak force and strain on the Achilles tendon; however, our findings indicate a divergent trend among elite athletes (11). All runners maintain a speed of 14 km/h during the half-marathon, which is a significant factor contributing to increased cumulative loading on the AT. The elite runners’ capacity to adapt to a high training volume (30), resulted in lower AT loading at the 10 km mark. The findings of this study suggest that half marathons can lead to increased AT accumulation, thereby elevating the risk of overuse injuries related to the AT. The PFJ peak stress, PFJ peak stress impulse, and cumulative weighted PFJ impulse were significantly increased during the treadmill half marathon. The increased contact time and step frequency can be attributed to the greater PFJ peak stress (66). Our findings are consistent with previous studies indicating that after fatigue running, PFJ stress significantly increases, which is associated with patellofemoral pain syndrome (PFPS) (11). Greater cumulative loading on the PFJ has been confirmed in cases of PFPS. Runners who develop PFPS have been shown to exhibit reduced knee flexion angles and longer contact times during the running stance phase (22).

The present study has several limitations that should be considered. Firstly, the limited sample size constrained the investigation of sex-specific effects. In the present study, we only compared biomechanical parameters at specific distance checkpoints (pre-running, 10 km, and 20 km) during a half marathon run, which limits generalizability to female or male runners. Furthermore, during the data collection, running shoes were not standardized, and both men and women ran at the same speed. Future studies should control for footwear and consider implementing sex-specific running speeds. Third, all trials were completed on a treadmill at a fixed speed. In the future, studies should consider investigating how long-distance running on different surfaces affects lower-limb biomechanics and injury mechanisms.

## 5 Conclusion

This study examined how treadmill half-marathons influence the lower extremity biomechanics of high-performance endurance runners. As the running distance increases, high-performing endurance runners show a significant decrease in ankle plantarflexion angle and ROM during the push-off phase. These results demonstrated that prolonged running induces foot plantar flexor fatigue and affects the running propulsion during the push-off phases. Furthermore, the knee flexion angle increased at 10 km and decreased at 20 km, which demonstrated the movement strategy to decrease the repetitive loading. Concurrently, a half marathon increases the AT and PFJ loading significantly at 20 km checkpoints. The findings demonstrated the importance of considering joint cumulative loading, particularly on the AT and PFJ during prolonged running.

## Data Availability

The raw data supporting the conclusions of this article will be made available by the authors, without undue reservation.
